# Automated Dermoscopy Image Analysis of Pigmented Skin Lesions

**DOI:** 10.3390/cancers2020262

**Published:** 2010-03-26

**Authors:** Alfonso Baldi, Marco Quartulli, Raffaele Murace, Emanuele Dragonetti, Mario Manganaro, Oscar Guerra, Stefano Bizzi

**Affiliations:** 1 Department of Biochemistry, Section of Pathology, Second University of Naples, Via L. Armanni 5, 80138 Naples, Italy; 2 Futura-onlus, Via Pordenone 2, 00182 Rome, Italy; E-Mail: raffaele@murace.it; 3 ACS, Advanced Computer Systems, Via della Bufalotta 378, 00139 Rome, Italy

**Keywords:** melanoma, dermoscopy, digital images, content-based image retrieval (CBIR)

## Abstract

Dermoscopy (dermatoscopy, epiluminescence microscopy) is a non-invasive diagnostic technique for the *in vivo* observation of pigmented skin lesions (PSLs), allowing a better visualization of surface and subsurface structures (from the epidermis to the papillary dermis). This diagnostic tool permits the recognition of morphologic structures not visible by the naked eye, thus opening a new dimension in the analysis of the clinical morphologic features of PSLs. In order to reduce the learning-curve of non-expert clinicians and to mitigate problems inherent in the reliability and reproducibility of the diagnostic criteria used in pattern analysis, several indicative methods based on diagnostic algorithms have been introduced in the last few years. Recently, numerous systems designed to provide computer-aided analysis of digital images obtained by dermoscopy have been reported in the literature. The goal of this article is to review these systems, focusing on the most recent approaches based on content-based image retrieval systems (CBIR).

## 1. Introduction

Human cutaneous melanoma, a deadly skin cancer that develops through the malignant transformation of melanocytes, can be a highly aggressive neoplasm, characterized by a rapidly growing incidence and an elevated mortality rate. Although early melanomas are locally controlled with surgical excision [1], up to 20% of patients will develop metastatic tumors due to its high capability to invade and rapidly metastasize to distant organs [2]. When human melanoma progresses to metastatic stage, powerful mechanisms to resist chemotherapy, radiation, and biological intervention are established in the neoplastic lesions, thus hampering the efficacy of current medical therapies [3,4]. Melanoma and non-melanoma skin cancers currently constitute one of the most common malignancies in the caucasian population, and the worldwide incidence and mortality rates are continuously increasing [5]. In particular, melanoma incidence has increased more than any other cancer, and currently has reached 18 new cases per 100,000 population per year in the United States [6]. Because advanced skin cancers remain incurable, early detection and surgical excision is currently the only approach to reduce mortality. The traditional screening tests require a skin naked-eye examination by an experienced clinician. One of the most widely used methods for evaluating pigmented skin lesions (PSLs) with the naked-eye is the ABCD rule [7]. However, this system may fail to detect many difficult or borderline PSLs that are small or/and regular in shape or color.

## 2. Dermatoscopy and Computer-Aided Analysis of Digital Images

Dermoscopy (dermatoscopy, epiluminescence microscopy, incident light microscopy, skin surface microscopy) is a non-invasive diagnostic technique for the *in vivo* observation of PSLs, allowing a better visualization of surface and subsurface structures (from the epidermis to the papillary dermis). This diagnostic tool permits the visualization of morphological structures of the epidermis an the dermo-epidermal junction until the papillary dermis, thus opening a new dimension in the analysis of the clinical morphologic features of PSLs. Several studies [7] have shown that this method may improve diagnostic sensitivity by 20–30% compared with clinical diagnosis by the naked eye. However, due to the complexity of patterns and their interpretation, the results of dermoscopic examination have limitations, especially for the inexperienced, and they are effective only if the user is formally trained. In order to reduce the learning-curve of non-expert clinicians and to mitigate problems inherent in the reliability and reproducibility of the diagnostic criteria used in pattern analysis, several indicative methods based on diagnostic algorithms have been introduced in the last few years. The ABCD rule, the 3-point checklist, the 7-point checklist, the Menzies’s method and the CASH algorithm are the most relevant ones [7,8,9]. All these dermoscopic criteria and diagnostic algorithms have been proposed and analyzed in recent years by an exponential number of publications [10] Recently, numerous systems designed to provide computer-aided analysis of digital images obtained by dermoscopy have been reported in literature. The aim of these systems is to transfer the ABCD attributes, as well as other characteristics based on texture, into automatically computed quantities, and use these parameters in order classify the PSLs [11]. The proposed computer-assisted methods differ from each other depending on the set of features extracted from the digitalized dermoscopic images, and on the feature selection and classification methods used. Despite multiple publications that assess the improved diagnostic accuracy of these instruments compared with that of clinicians, the effectiveness of these systems depends largely on the dataset used [12,13]. Indeed, most of the studies were not comparable because of lack of image standardization. Moreover, the use of restricted and selected databases made the systems applicable only in experimental conditions. To note, a recent multicenter study conducted in 13 European dermatologic centers validated the performance of a diagnostic and neural analysis of skin cancer [14]. This experimental approach took advantage of artificial neural networks to analyze a large database of dermatoscopic images, reaching a diagnostic accuracy quite similar to dermatologists. 

A different experimental approach to demonstrate the feasibility and reliability of automated analysis of dermoscopic images in melanoma diagnosis has been described by Burroni *et al.* [15], using two different statistical classifiers in two different dermatologic units. On the other hand, the group of Gerger [16] has defined a different strategy, combining a tissue counter analysis with machine learning algorithms in automated epiluminescence microscopy for differentiating between malignant and benign PSL. Another preliminary observation by Oka *et al.* [17] has defined a linear discriminant analysis of dermoscopic parameters. Finally, the study of Seidenari *et al.* [18] defined a method for automatic color evaluation of dermoscopic images.

In conclusion, all these studies have shown that the currently proposed image analysis systems are able to identify correctly the clinically obvious melanomas, but they have limited capabilities to discriminate between borderline lesions and early malignant melanoma [19]. Therefore, so far, these computer-assisted diagnostic imaging tools provide little benefit for the experienced clinician. An experienced clinician bases her/his decision on experience, as well as on complex inferences and extensive knowledge, which is very difficult to mimic in a mathematical algorithm.

## 3. Medical Images: Toward a Digital Database

The convergence of the biological and information sciences is currently producing a paradigm shift in health care. Electronic processing of medical data has opened many possibilities for improving medical tasks such as diagnosis, surgical planning or therapy, both in daily clinical practice and clinical research. Medical diagnosis and intervention increasingly relies upon images, of which there is a growing range available to the clinician: X-ray (increasingly digital, though still overwhelmingly film-based), ultrasound, MRI, CT, PET scans, *etc*. More than patient data, the medical images by far represent the major amount of information collected for medical data. Picture Archiving and Communication Systems (PACS) deployed in hospitals today address some of the challenges related to medical data management. Yet, the possibility of aggregating the information stored in the imaging data to provide assistance for diagnostic activities is still not at all exploited, as medical practitioners still rely on atlases for support. With the advent of digital images, the production of digital atlases has become possible. Nevertheless, to provide the maximum benefit to diagnostic and analysis activities that rely on them, large digital atlases need to be effectively navigated in terms of content, providing the practitioner with just the information that most relates to the cases under examination. Rather than just relying on textual patient information or on subjective and error-prone annotations describing the images, this casting of generic image content into a summary of the atlas - in terms of the specific issues being analyzed - needs to be performed based on the actual contents of the data resulting from the imaging procedure.

## 4. Content-Based Image Retrieval (CBIR) Systems

An image retrieval system is part of a digital asset management system for browsing, searching and retrieving images from a large database. Digital asset management is concerned with the ingestion, annotation, cataloging, storage, retrieval and distribution of assets such as photographs, animations, videos and music. Generally, the "asset" being managed is constituted of a binary stream of data that can be stored in a file and that represents actual content that can be, for instance, visualized or listened to. As an addition to the data stream itself, the asset is accompanied by its "metadata". Metadata is a description of the asset that can detail among others asset content (what is in the package?); the means of encoding/decoding (e.g., JPEG, MPEG 2); provenance (history to point of capture); ownership; rights of access. While metadata content can be quite extensive, it is not meant to represent a complete description of the contents of the data stream in textual terms. Its main purpose is instead normally to capture information about the context of the acquisition.

Most traditional and common methods of image retrieval utilize some method of adding metadata such as captioning, keywords, or descriptions to the images so that retrieval can be performed over the annotation words. Manual image annotation is time-consuming, laborious and expensive; to address this, there has been a large amount of research done on automatic image annotation: image meta search is a type of search engine specialized on finding pictures, images, animations *etc*. Like text search, it is designed to help users find information in large archives using keywords or search phrases sorting results by relevancy.

A common misunderstanding when it comes to image search is that the technology is based on detecting information in the image itself, whereas most image search works as other textual search engines. The metadata of the image is indexed and stored in a database, and when a search query is performed the image search engine looks up the index, and queries are matched with the stored information. Some search engines can automatically identify a limited range of visual content, e.g., faces, trees, sky, buildings, flowers, colors, *etc*.

CBIR intends overcoming the limitations inherent in metadata-based systems: textual information about images can be easily searched using existing technology, but requires humans to personally describe every image in the database. This is impractical for very large databases, or for images that are generated automatically. It is also possible to miss images that use different synonyms in their descriptions. Systems based on categorizing images in semantic classes like "cat" as a subclass of "animal" avoid this problem, but still face the same scaling issues. Without the ability to examine image content, searches must rely on metadata such as captions or keywords, which may be laborious or expensive to produce. In contrast, CBIR is the application of computer vision to the image retrieval problem. "Content-based" means that the search will analyze the actual contents of the image. The term 'content' in this context might refer to colors, shapes, textures, or any other information that can be derived from the image itself. A parallel field is that of content-based video indexing and retrieval and automatic annotation: similar issues and objectives are shared, while the video domain has specific issues of its own as well as specific possibilities to exploit additional information related to the temporal dimension of the video stream.

## 5. CBIR Research Literature Review

A large volume of scientific research has been produced in the last 20 years about CBIR, starting around February 1992 when the US National Science Foundation organized a workshop in Redwood, California, to identify major research areas that should be addressed by researchers for visual information management systems that would be useful in scientific, industrial, medical, environmental, educational, entertainment, and other applications [20]. The techniques, tools and algorithms that are used originate from fields such as statistics, pattern recognition, signal processing, and computer vision.

Two central review papers have been published in the literature about the subject.

The first one dates to the year 2000 [21] and reports early progress in the field. The paper presents a review of 200 references in CBIR. It starts with discussing the working conditions of content-based retrieval: patterns of use, types of pictures, the role of semantics, and the sensory gap, that is the key issue of linking high-level user interests in terms of domain-specific applications to the low level signals acquired by an imaging instrument. Subsequent sections discuss computational steps for image retrieval systems. Similarity of pictures and objects in pictures is reviewed for each of the feature types (including color, texture, structure), in close connection to the types and means of feedback the user of the systems is capable of giving by interaction. Aspects of system engineering are discussed in the review paper: databases, system architecture, and evaluation. The authors present their view on the many different aspects of CBIR as a research domain: the driving forces of the field, the heritage from and influence on the companion domain of computer vision, the role of similarity and of interaction, the need for databases, the problem of evaluation, and the role of the semantic gap. After discussing the heritage of computer vision research, applications of content-based retrieval are analyzed considering three broad types: target search, category search, and search by association. Target search focuses on retrieving "signs", specific kinds of single objects for which well identified detection strategies exist. This kind of search builds on pattern matching and object-recognition. Category search is instead about identifying and retrieving all images that may be associated with the characteristics of a broader group or set of acquisitions tied together by broader low-level similarity criteria. The association process is about high-level, semantic application domain terms to be used for screening a large image database. It necessarily includes some type of user interaction and is essentially iterative, interactive, and explicative. Therefore, association search is hampered most by the semantic gap. Dataset display and result ranking relevance feedback has to be understood by the user, so the emphasis must be on developing features transparent to the user. The need for databases is discussed too. When data sets grow in size and when larger data sets define more interesting problems, both scientifically as well as for the public, the computational aspects can no longer be ignored. Parallel, distributed processing options need to be evaluated and put to effective use to obtain a level of performance suitable for real world applications. The problem of evaluation is also considered: the quality of the results returned by a search/ranking system needs to be measurable for quantifiable progress to be made in the field. The paper notes that the aim of content-based retrieval systems should be to provide maximum support in bridging the semantic gap between the simplicity of available visual features and the richness of the user semantics. Significantly, the authors conclude that one way to resolve the semantic gap comes from sources outside the image, by integrating external information about the image in the query. Prior/ context knowledge about an image can come from a number of different sources: the image content, labels attached to the image, images embedded in a text, *etc*. 

The second fundamental review paper in CBIR dates to 2008 [22] and reports current trends about the field. In this article, the authors survey almost 300 key theoretical and empirical contributions in the 2000-2008 period related to image retrieval and automatic image annotation, and in the process discuss the spawning of related subfields. They also discuss significant challenges involved in the adaptation of existing image retrieval techniques to build systems that can be useful in the real world. Further analysis is presented on the impact of image retrieval on merging interests among different fields of study, such as multimedia, machine learning, information retrieval, computer vision, and human-computer interaction. The authors note that while aspects including systems, feature extraction, and relevance feedback have received a lot of attention, application-oriented aspects such as interface, visualization, scalability, and evaluation have traditionally received lesser consideration although they should also be considered as equally important. With the advent of very large-scale images, biomedical and astronomical imagery have become typically of high resolution/dimension and are often captured at high throughput, posing yet new challenges to image retrieval research. A long-term goal of research should therefore also include the ability to make high-resolution, high-dimension, and high-throughput images searchable by content. 

The many issues related to the unsatisfactory performance of general-purpose CBIR and the objective of object recognition are addressed among others in [23]. The key problem to be solved is that the preliminary problem of context inference starting from the signal level has to be addressed before any effective similarity ranking can be carried out. Yet, making this preliminary selection of the correct application domain from the image signal alone is essentially unfeasible with the current state of the art. If the context is instead well known, then the search problem can be regularized by taking into account prior knowledge about the domain, as in the stringent case of computer vision/ image search for parts monitoring e.g., on a conveyor belt in an industrial environment. 

The added simplicity produced by prior knowledge about the context of search is the fundamental reason why specialized CBIR systems are in widespread use in real-world environments ranging from biometrics (the IAFIS fingerprint matching system in use at FBI) to industrial applications. CBIR represents an effective solution, especially in cases in which the similarity evaluation between the images depends on the measurement of small details (as in the case of fingerprint minutiae) or of quantities that are difficult for human observers to quantify (e.g., skin texture in the case of dermatology). The steady increase in processing power and the inherently parallel nature of the problem render instead less a limiting factor than in the past any near real time requirements.

A further issue is the need to take into account application-domain terms in the analysis, effectively bridging the gap between the plain image signal level (the pixel values) and the higher level semantic one. This is both an applicative necessity and a possibility for regularizing the visual signal analysis with the structure inherent in human language.

A number of review articles have been published about CBIR in specialized domains and the issues they present. Specific attention has been paid to the domain of medical CBIR [24] and the integration of automated search in PACS in actual hospital institutions [25].

## 6. Existing Systems and Real World Applications

The large volume of scientific research produced on the topic of CBIR is unfortunately not paralleled as of 2010 by an equally significant availability of actually operational tools. Nevertheless, a number of systems for general-purpose CBIR have been developed over the last ten years. A significant subset of these can be used via the Internet for testing purposes (including Google Images [26], Microsoft Bing Image Search and a number of others [27]). A few other systems are available in the free software/open source domain [28,29] and can be used to categorize user content on local machines. Generic reverse search is also an active real-world application of CBIR: an example image is used to start a monitoring activity on the web for similar ones for copyright infringement issues. 

As for domain-specific CBIR, functionality is for instance included in a number of products for picture acquisition and management (Apple iPhoto, [30,31]) in the form of face recognition and retrieval [32] functions. CBIR for on-line shop navigation is also developing [sites such as "like.com" and "shopachu.com"]. An intermediate situation is the one in which well controlled acquisition procedures are carried out on natural scenes with high variability. In this category, a specific domain where inroads have been made in the last 10 years is that of CBIR for remote sensing images [33]. The applicability of prior information and precise direct models of the acquisition process seems in this case able to provide an effective regularization to the image search problem. A fundamental limit is in this case reached with new satellite systems able to operate at the metric level, providing what are in effect high resolution pictures of complex environments with much higher variability than before [34]. The context-inference problem reappears in this case in all of its complexity and system performance degrades accordingly.

## 7. Dermoscopic CBIR: The FIDE System

The process of describing picture content in textual terms takes on a specific meaning in the case of image-based diagnostic activities e.g., in dermatological settings. Documenting this image interpretation process for repeatability, educational as well as legal issues is essential to reducing diagnostic errors and improving effectiveness at different levels of medical organizations.

Although their significance is specific to the applicative setting, the techniques that can be employed to answer this need bear a broader meaning related to the well-established, general field of digital media asset management. Automatic image-based diagnosis attempts have been the subject of active research in biomedical image analysis for a number of years [29,35,36,37,38].

Abstract features and machine learning methodologies or problem oriented model-based systems have been employed. Yet, the main problem with automated diagnostic analysis is the disproportionate cost of false negatives. The role of the expert interpreter and the related responsibilities should not be abdicated.

The approach of our CBIR system for dermoscopic images, that is FIDE [39], is to document the image analysis side of the diagnostic process, focusing on accompanying and aiding it and on providing efficient digital atlas navigation aiming both at providing precision (cases most similar to the one under analysis) and clarifying context (similar cases in different categories). Instead of getting indications on a possible diagnosis by an automated interpretation system, the doctor needs to be recognized in her/his role and aided by an efficient search system able to present a number of similar cases from a large atlas. A CBIR system can be used to retrieve and display cases that are objectively similar by image content to the one under analysis, together with medical records for the analyst to consider in order to document and assist the interpretation and diagnosis procedures. Furthermore, the diagnostic procedure can be documented by logging the acquired images.

In the FIDE system, the statistical analysis performed in order to assess the similarity among image items is based on a hierarchical Bayesian model-based data analysis approach. The RGB signal level model p(D) is linked to a high-dimensional primitive descriptor level p(P) taking into account color as well as geometric information extracted from the data and in turn to a secondary, lower-dimensionality, independent synthetic descriptor level p(S) by conditional probabilistic links p(P|S), p(S|P). Inference is conducted in order to obtain the posterior density p(S|P) p(P|D) p(D). The obtained densities are compared with each other taking into account a bank of distance and divergence measures to carry out an association level search procedure. Category search is employed to limit search results discriminating among retrieval outcomes of different natures. Standard measures of retrieval performance that are considered in the development and evaluation of the system performance. Clusters are defined by the available supervised diagnoses attached to each of the items. The centers of mass of each of the clusters can be calculated, as the relative distances of the different centers of mass provide a further a measure of the quality of the ranking output by the retrieval system.

The FIDE system is effective in retrieving PSL images with known pathology visually similar to the image under evaluation giving a valuable and intuitive aid to the clinician in the decision making process. Indeed, we argue that a system, able to retrieve and present cases with known pathology similar in appearance to the lesion under evaluation, may provide an intuitive and effective support to clinicians which potentially can improve their diagnostic accuracy. In addition, this CBIR system can be useful as a training tool for medical students and inexpert practitioners given its ability to browse large collections of PSL images using their visual attributes. The system will allow the user to mark retrieved images as positive and negative relevance feedback. This very important feature will permit both to better evaluate the performance of the proposed system and, consequently, to further tune the weighting factor parameters in order to improve the relevance of the retrieved PSL images. Furthermore, the proposed system can be used to create appropriate CBIR Web Services that can be used remotely to perform query-by-example in various PSL image databases around the world and can be a very good complement to text-based retrieval methods. 

## 8. Conclusion

**Figure 1:**
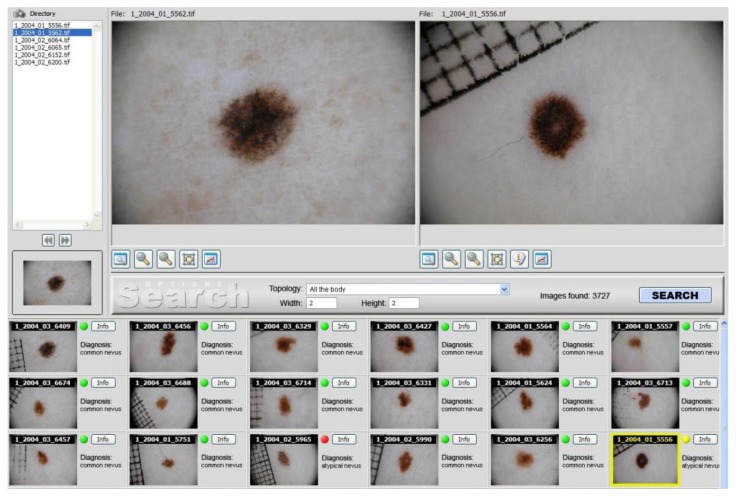
FIDE graphical user interface. The query image on the upper left is compared to those in the archive. Most similar results by image content are returned in the lower thumbnail pane together with their respective biopsy diagnoses (red dots for unfavorable outcomes, green ones for normal ones). Single images chosen from the returned set can be visually compared with the query by double-clicking their thumbnails, loading their original in the view panel on the upper right.

Dermoscopy actually has higher discrimination power than naked-eye examination to detect melanoma, if performed by trained physicians. Computer-assisted automated diagnosis of PSL by means of CBIR systems is a promising research field, that probably will change the future management of skin tumors. FIDE represents, indeed, the first CBIR system successfully applied to dermoscopic images (Figure 1). The installation of the described system at several medical centers is crucial in order to assess the clinical impact when it is used in clinical practice. Finally, a similar search engine finds possible usage in all other sectors of imaging diagnostic, or digital signals (NMR, Video, Radiography, Endoscopy, TAC, *etc*.), which could be supported by the huge amount of information available in medical archives.
